# Panoramic radiographic study of mental foramen in selected dravidians of south Indian population: A hospital based study

**DOI:** 10.4317/jced.52174

**Published:** 2015-10-01

**Authors:** Vaibhav Gupta, Parag Pitti, Amar Sholapurkar

**Affiliations:** 1 BDS, MDS, Post Graduate student, Department of Public Health Dentistry, M.S. Ramaiah Dental College and Hospital, MSRIT Post, MSR Nagar, Bangalore – 560054, India; 2 BDS, Apex Dental Clinic, A - 204, A wing, Jain Apartment, 60 feet road, Bhyander West, Thane – 401101, India; 3 BDS, MDS, FAGE, Lecturer, Clinical Dentistry and Oral Radiology, College of Medicine and Dentistry, James Cook University. Cairns, Queensland - 4878, Australia

## Abstract

**Background:**

This study aimed at documenting information on appearance, size, horizontal and vertical locations of Mental Foramen (MF) in Panoramic Radiograph. We also analyzed the age and gender differences with radiographic appearance and location of MF. We evaluated these findings in our population and co-relate with results of previous studies.

**Material and Methods:**

1662 panoramic radiographs were evaluated, of which 245 fulfilled the inclusion criteria. Each radiograph was traced to record the horizontal and vertical locations. The size of MF was recorded using digital caliper and its appearance was determined by visual examination. Chi-square and t-test were employed.

**Results:**

The most common appearance of MF was continuous type and the tests showed significant difference with age and gender. The most frequent horizontal location of MF was “location c” with no statistical significant difference with age and gender. The MF was most commonly positioned mesially in relation to the apex of second premolar with no significant differences with gender. The vertical location of the foramen varied drastically with no statistical significant difference in both sides. The difference in dimensions on the left and right sides were not statistically significant.

**Conclusions:**

Determining the morphological appearance and positional variation of MF is important for isolation of mental nerves and vessels when administering local anesthesia and performing surgeries. We therefore stress the importance of accurate radiographic identification of MF and interpretation. Our research findings can be used as reference material by the dental practitioners of South India while performing clinical procedures that involve MF.

** Key words:**Mental foramen, mental nerve, panoramic radiograph, mandible.

## Introduction

The Mental Foramen (MF) is an important anatomical structure located in the body of mandible. It represents the termination of mental canal which opens onto the surface in oblique direction. The mental bundle passes through MF and supplies sensory in-nervations and nutrition to the chin, lower lip and gingiva on the ipsilateral side of the mandible (1). As there are no absolute ana-tomical landmarks for reference and the foramen cannot be directly visualized or palpated, radiographic evaluation of the position of MF becomes an obligation for accurate diagnosis (1).

The accurate identification of location of MF is important for both diagnostic and clinical procedures. The mental nerve exiting the MF usually has three to four branches for innervation of the soft tissues of the chin, lower lip, facial gingiva and mucosa in the anterior mandible. The clinician is advised to observe a safety distance when performing incisions and osteotomies in the vicinity of the MF. The mental bundle could be traumatized during periapical surgery, orthognathic surgery, mandibular fixation/reduction resulting in paresthesia or anesthesia. Additionally, local anesthesia of the terminal incisive branches of the inferior alveolar nerve and mental nerve can be obtained if the MF is located near it. Also recent development of mandibular implant technique and increasing frequency of orthognathic surgery has increased the possibility of surgical procedures near the MF.

On radiographs, MF appears as a radiolucent area in lower premolar region, sometimes overlapping the apex of a premolar (2,3). Its visualization on intraoral radiography may be difficult. The most common difficulty is that its position is below the edge of the film. Patient with the small mouth, large mandibular tori and a shallow floor of mouth or malposed teeth may prevent the proper placement of film during radiographic examination. Cases such as these may require a different radiographic technique to visualize the foramen. Moreover, due to the oblique direction of mental canal in the mesiodistal and inferosuperior planes, it cannot always be observed in a periapical radiograph (4).

Panoramic radiograph has gained popularity in the last three decades. The advantages of this technique over intraoral radiography include a great area of soft and hard tissue coverage, continuity of the visualized area, and the rapidity with which the view is formed. The ability to view the entire body of mandible allows a more accurate localization of MF in both horizontal and vertical directions. However it appears slightly larger on panoramic radiographs than on the periapical radiographs (4) because of magnification. However correction of the measurement value to 100% can be done by using the magnification factor provided by the manufacturer.

The MF has been reported to vary in its appearance, size and locations in different population groups (5-7). Moreover, dental practitioners have been experiencing problems during injections and operative procedures involving MF as the mental foramen is frequently encountered in a number of maxillofacial surgical procedures (7). Hence this study aimed at documenting anatomical information on appearance, size, horizontal and vertical locations of MF in Panoramic Radiograph. We also determined the relationship of age and gender with its radiographic appearance and location. With a thorough literature search in PUBMED/MEDLINE and to the best of our knowledge, till date, there has been no such comprehensive study representing the Dravidian ethnic group of South India. This study was done to evaluate these differences in our population and co- relate with the results of other studies.

## Material and Methods

The study population included those patients who attended Oral Medicine and Radiology department, Manipal College of Dental Sciences, Manipal, India, from January 2011 to December 2012 and received treatment that required an Orthopantomogram (OPG). Panoramic radiographs of patients radiographed in the Radiology section within this time period were retrieved from the Medical records section and retrospectively evaluated. Clear panoramic radiographs of high quality and good visibility of anatomic structures for measurements were only included in the study. Sample size was calculated using the formulae 4pq/L2, where *p* = Proportion of the patients with MF between first and second premolar which was 0.47 based on the previous study (8). q = 1-p = 0.53 and level of precision (L) was considered to be 6.4%.

Patients aged from 19-65 years were divided into 2 groups. Group A consisted of those patients between 19 and 40 years and Group B were between 41 and 65 years of age. Informed consents were taken from the patients before the study was conducted. Patient’s radiographs were then randomly selected and examined for eligibility against selection criteria. 1662 panoramic radiographs were evaluated out of which 245 fulfilled the inclusion criteria. Since the reliability of panoramic radiography technique for imaging the mandible is highly dependent on the patient’s head position (while taking the OPG), these radiographs were selected by those taken by experienced radiographer using the same panoramic unit. The radiographs (Eastman Kodak company, Rochester, NY, USA) were taken using PLANMECA 2002EC (PROLINE, Finland) considering standard exposure parameters (68kv, 8Ma, 18 sec, total filtration 2.5mm Al, focal spot .3mm and magnification factor 1:1.2) and processed using an automatic processor(Kodak X-OMAT 3000RA, Eastman Kodak company).

The inclusion criteria were as follows:

1. Presence of all mandibular teeth between right first molar and left first molar.

2. Mental foramen was clearly discernable at least on one side.

3. Permanent teeth were fully erupted.

4. First and second premolars were in reasonably normal position and alignment

The exclusion criteria were as follows:

1. Patients below 19 years of age

2. Presence of severe crowding and spacing in lower arch

3. Missing upper premolars because of possibility of over eruption of lower premolars

4. Presence of radiolucent lesion in the lower jaw anywhere in the area extending from right first molar to left first molar.

5. Presence of periodontal lesions.

6. Patients undergoing/already underwent orthodontic treatment.

7. Fracture line involving the parasymphyseal region.

To ensure consistency, one investigator was responsible for selection of radiographs based on the inclusion and exclusion criteria. The radiographs were observed in a dimly lit room on a masked view box by two trained investigators and later verified by the chief investigator by random selection. Each radiograph was traced(using a 0.3 mm pencil) on an acetate paper sheet (Ultraphan, St. Paul, MN, USA) to record the horizontal location, vertical locations and size of MF. The appearance of MF was determined by visual examination.

The appearance of mental foramina on the panoramic radiograph was classified as any one of the four different types (9-12).

1. Continuous type: foramen which showed continuity with the mandibular canal.

2. Separated type: foramen which was distinctly separated from mandibular canal.

3. Diffuse type: foramen which had an indistinct border.

4. Unidentified type: the foramen which could not be identified on the panoramic radiographic under given exposure and viewing conditions.

If there appeared to be multiple foramina, then the uppermost and the nearest landmark to the mandibular canal was considered as true radiographic MF.

The horizontal location in relation to the apices of the teeth were determined and categorized (9,10,13) as follows.

a. Anterior to first premolar

b. In line with first premolar

c. Between first and second premolar

d. In line with second premolar

e. Between second premolar and first molar

f. In line with first molar

The long axis of the premolars and 1st molar were considered as vertical references to determine the horizontal location.

**Figure 1:**
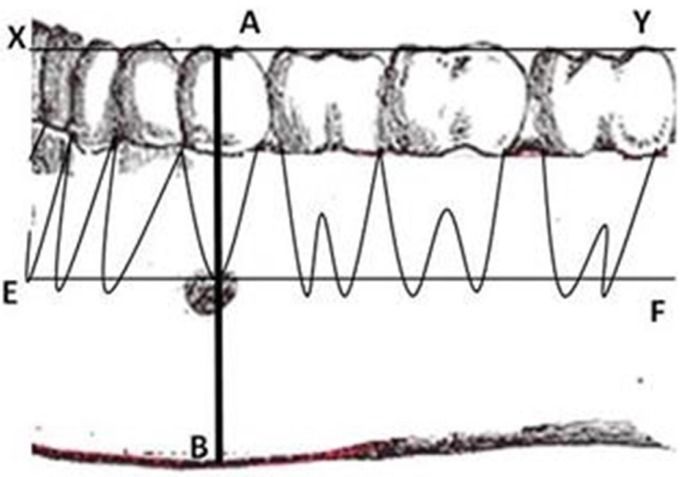
Determining the average position of mental foramen on panoramic radiograph. A horizontal line XY was drawn at the occlusal level. Another line EF was drawn parallel to the line XY at the apex of second premolar. Perpendicular line AB was drawn passing through the apex of the mandibular second premolar through the long axis of the clinical crown (perpendicular to lines XY and EF) to the inferior border of the mandible. The average position of the MF (in relation to the line AB) relative to the apex of 2nd premolar was recorded as mesial, distal or intersecting this line.

The average position was determined as shown in figure 1. A horizontal line XY was drawn at the occlusal level. Another line EF was drawn parallel to the line XY at the apex of second premolar. Perpendicular line AB was drawn passing through the apex of the mandibular second premolar through the long axis of the clinical crown (perpendicular to lines XY and EF) to the inferior border of the mandible. The average position of the MF (in relation to the line AB) relative to the apex of 2nd premolar was recorded as mesial, distal or intersecting this line.

**Figure 2:**
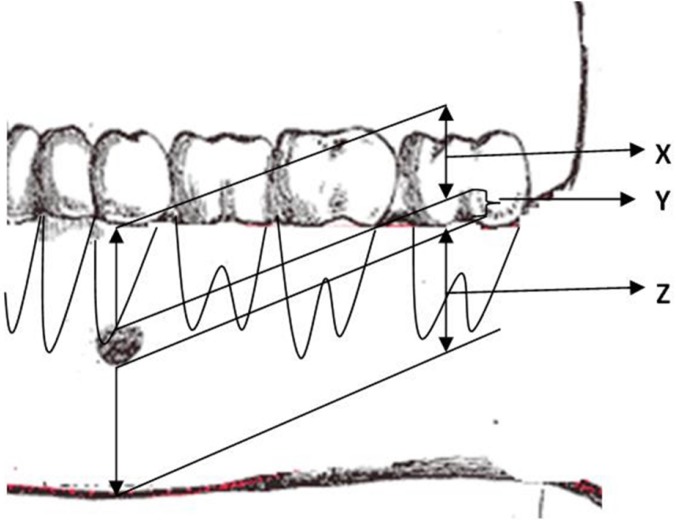
Determining the Vertical location of mental foramen on panoramic radiograph. Definition of values of “x,””y,” and “z” as used to determine relative vertical location of mental foramina. X: From the alveolar ridge to the upper border of mental foramen Y: The diameter of mental foramen itself Z: From the lower border of foramen to the lower border of mandible The ratio of x:z will give the relative vertical field of mental foramen.

The vertical location was estimated (11,12) by determining the shortest perpendicular line joining the alveolar ridge and the lower border of mandible, passing through the center of MF. Measurements were made (in mm) as shown in figure 2 i.e. from the alveolar ridge to the upper border of MF (x), from the lower border of foramen to the lower border of mandible (z), the diameter of MF itself (y). The ratio of x:z gave the relative vertical field of the foramen. Size of each MF was recorded in mm both in horizontal and vertical direction using digital caliper (series 727, starret, Itu, SP, Brazil). Magnification compensation was performed using the factor (20%) provided by the manufacturer.

The Intraclass correlation coefficient and Kappa statistics for the inter and intra examiner reliability were 0.82 and 0.95 respectively. Data entry and statistical analysis were carried out with the statistical package for social sciences (SPSS/PC for Windows, version 19.0, SPSS Inc.,Chicago, IL, USA). Descriptive and analytical statistics were derived. Chi-square and t-test were employed. The level of two-sided significance was set at 5%.

## Results

1662 panoramic radiographs were evaluated out of which 245 fulfilled the inclusion criteria. The radiographs were those of 156 males and 89 females in the age range of 19 and 65 years. The appearance of MF was found to be “Continuous” type in 34.3% males and “Separated” in 33.1% females ( Table 1). However in both age groups (Group A and Group B) majority of MF appeared to be continuous type in 28.1% and 37.3% cases respectively. The test results however showed a significant association of age (*p* = 0.0004) and gender (*p* =0.006). The most common horizontal location of MF was found to be Location “c” accounting to be 39.9% and 39.1% in males and females respectively ( Table 2). Location “c” was again commonest among both the age groups with 38.6% cases in Group A and 42.1% cases in Group B ( Table 2). However there was no significant association of age (*p* = 0.841) and gender (*p* = 0.767) with the horizontal location of MF. The average position of MF relative to the apex of second premolar ( Table 3) was found to be 47% on the mesial side, 34.7% on the distal side and 18.3% intersecting with the apex of respective second premolar. However there was no statistical significant association with gender (*p* = 0.910 for male, *p* = 0.055 for female). The vertical location of MF ( Table 4) was found to vary on left and right sides (X:Z = 1.34+0.99 on left and 1.48+1.12 on the right). The results of t test were not significant. The average horizontal dimensions of foramen on right and left sides were 2.61±1.83mm and 2.81+1.71mm respectively. The average vertical dimensions of foramen on right and left sides were 2.24+1.55mm and 2.29+1.39mm respectively. The difference between Horizontal and vertical diameter on the left and right side was not statistically significant.

**Table 1:**
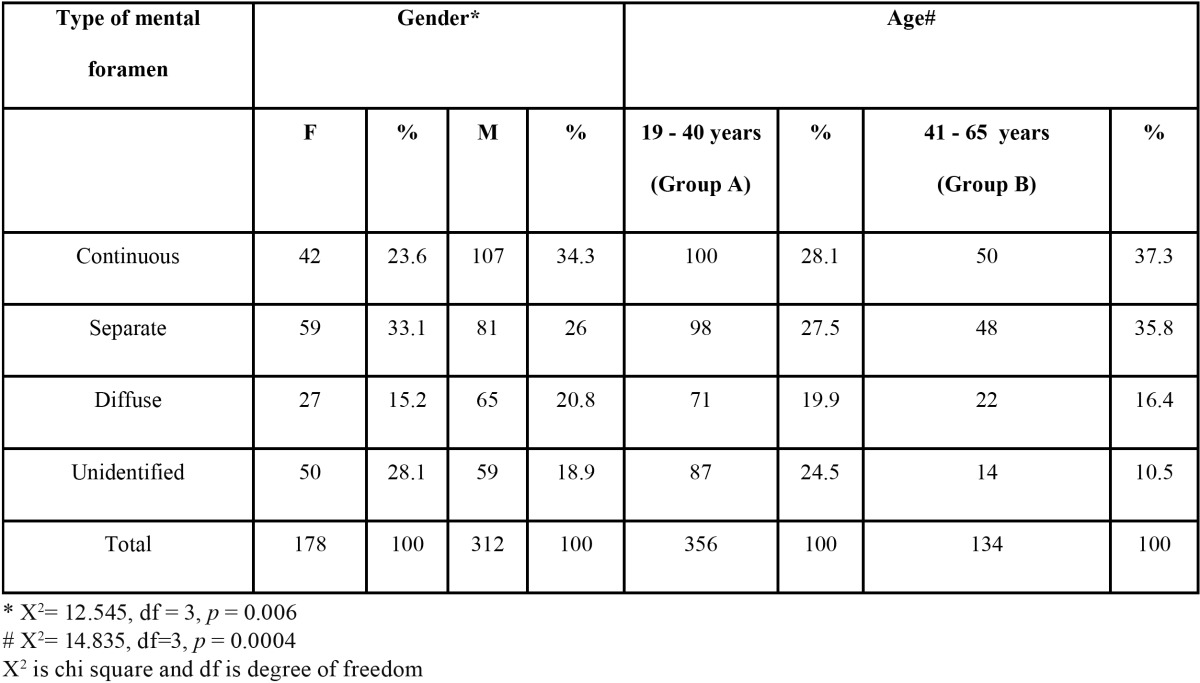
The appearance of mental foramina on panoramic radiograph bilaterally.

**Table 2:**
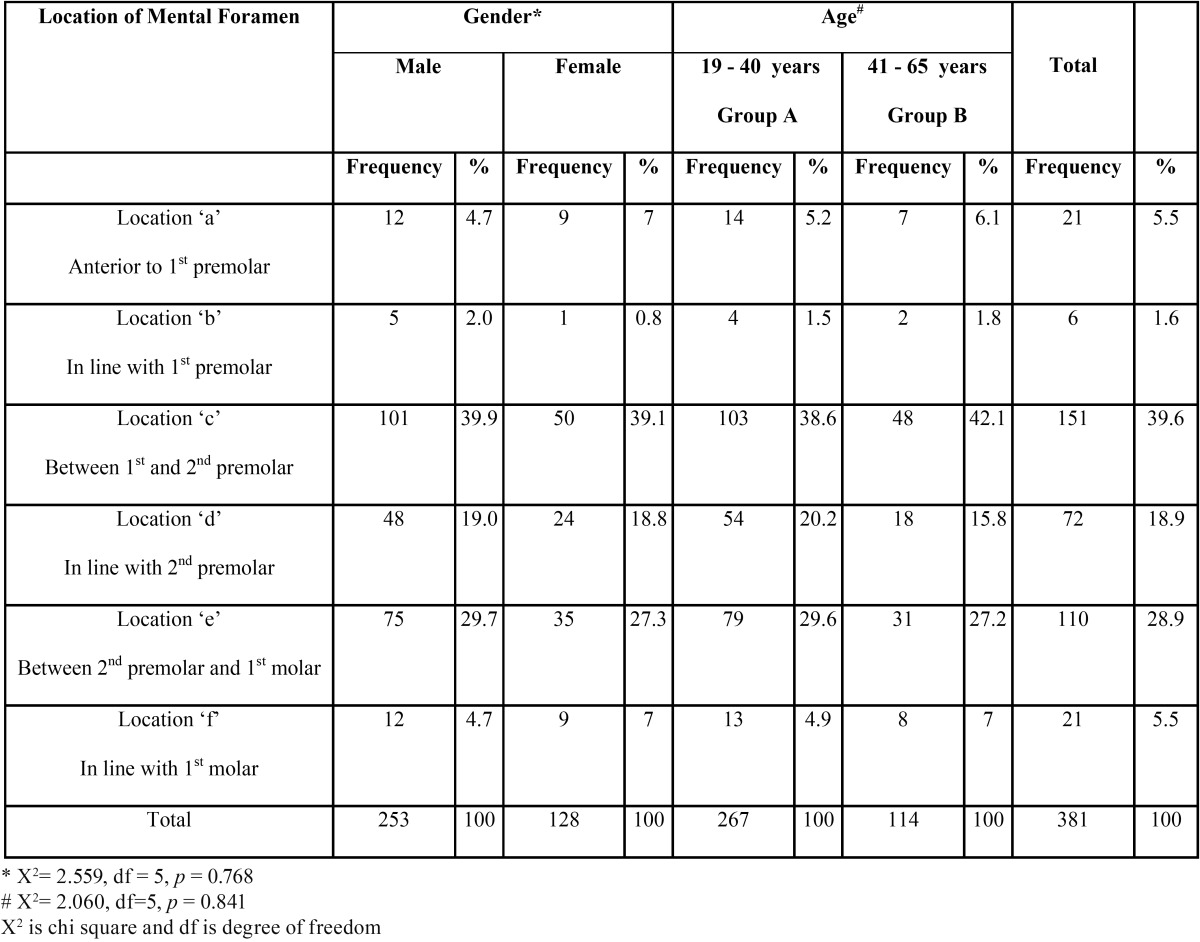
Frequency of horizontal location of mental foramen (in relation to the apices of the teeth on the panoramic radiograph).

**Table 3:**
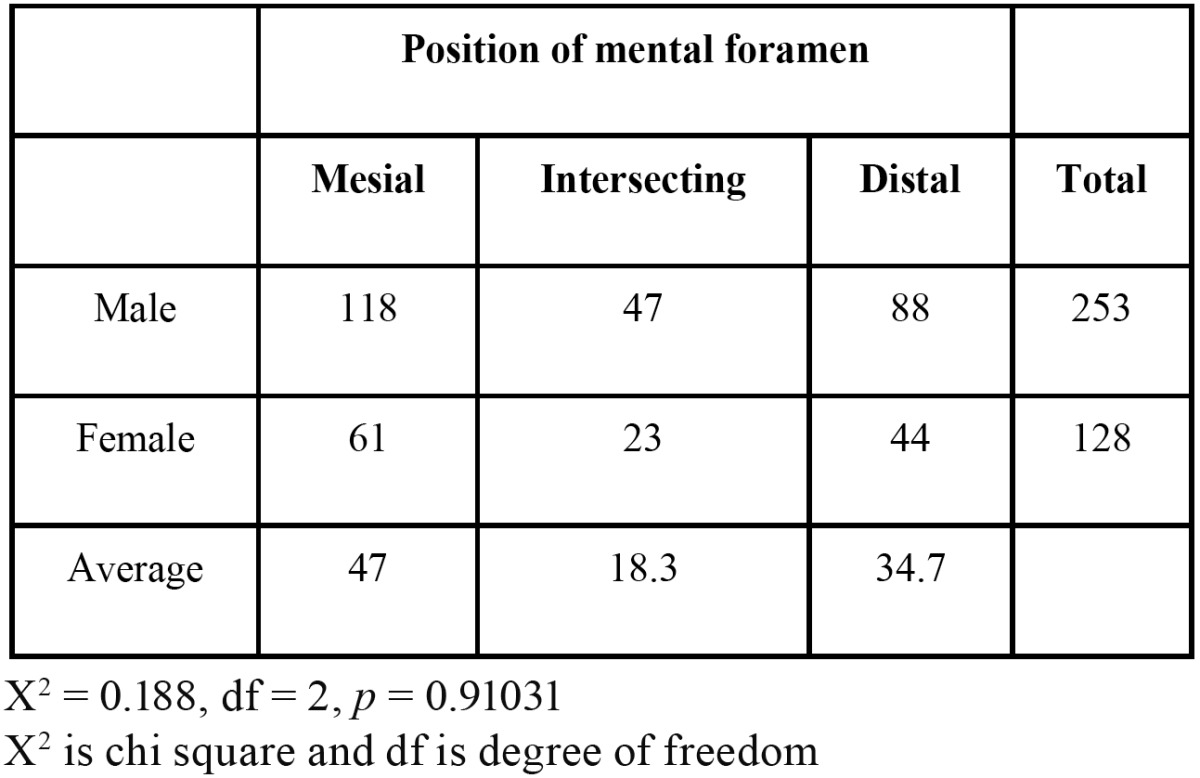
Average position of mental foramen relative to the apex of second premolar.

**Table 4:**
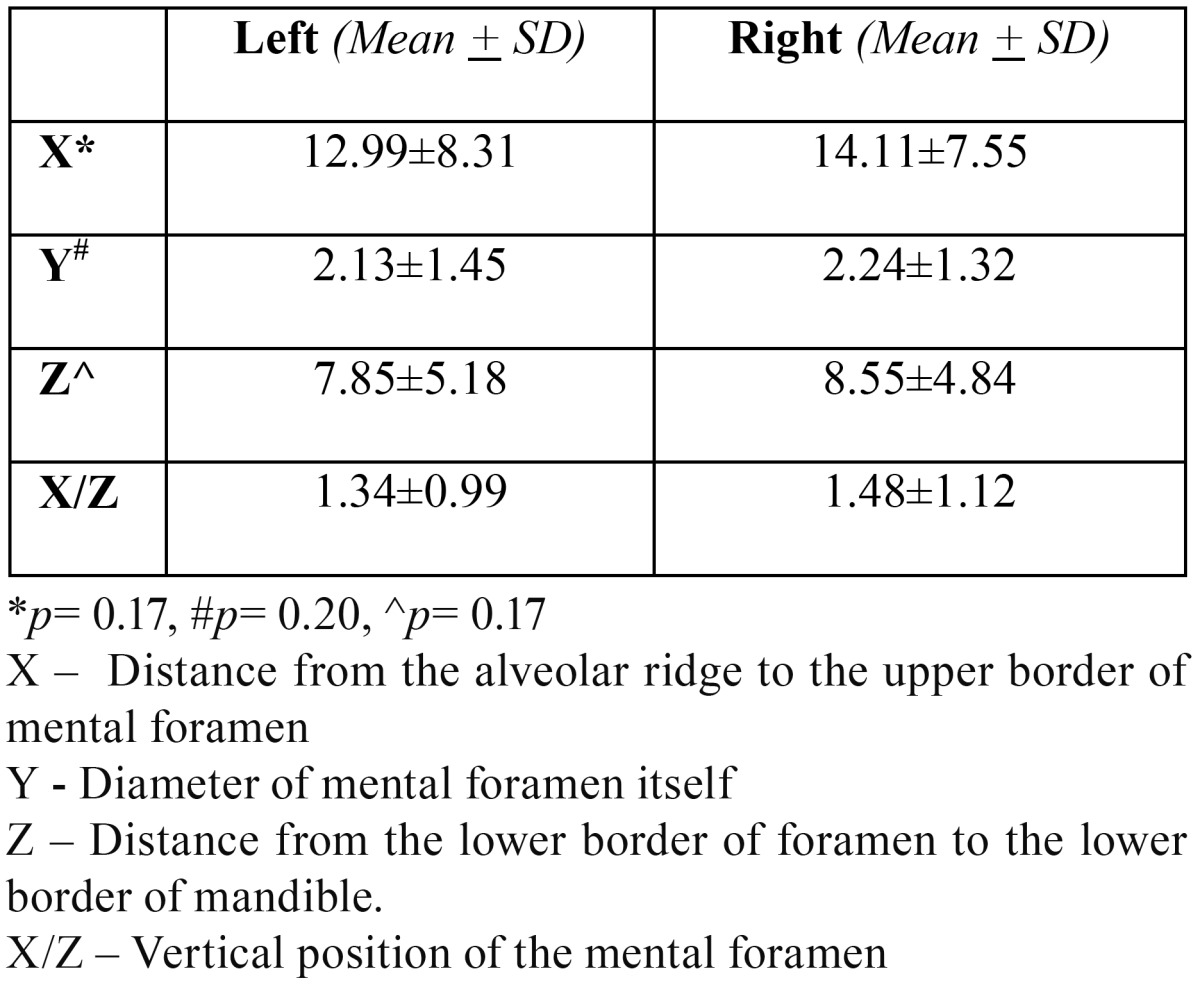
Vertical Location of mental foramen (mm).

## Discussion

Although it is clear from previous studies that position of mental foramen is the pre requisite for any mandibular anterior surgical procedure, there are significant differences between different populations (5-7). Hence, it is important to know these differences anatomically and statistically and our study has shown the same in South Indian population. Edentulous, periodontally weak cases, patients with history of orthodontic treatment and cases with fracture line in symphysis and parasymphysis region were not included in this study. Full complement of permanent dentition from right first molar to left first molar was selected as criteria for inclusion. This was done in order to avoid any changes in appearance of position of foramen due to changes in bone height or drifting of adjacent teeth into edentulous region. This study overcomes drawbacks of using Intraoral Periapical (IOPA) for determining the position of mental foramen as done by Fishel *et al.* (14). Ability to view entire mandible and follow the course of inferior alveolar and mental canal using fixed angulations and exposure makes it more reliable for tracking mental foramen. Youse *et al.* (11,12) evaluated 297 patients, and reported that the most frequent appearance was separated (43%), followed by diffuse (24%), continuous (21%), and unidentified (12%) whereas in our study most frequent appearance was continuous (30.4%) followed by separated (28.6%), unidentified (22.2%) and the diffuse (18.8%) variant respectively.

In previous studies(1,3,4,9,13,15,16) the most common horizontal location of mental foramen was found to be in line with the longitudinal axis of second premolar, where as in our study, majority of MF were located between first and second premolar which is analogous to the findings by other studies (2,8,10,14). Hence it is clear that location of mental foramen varies with different population. No significant gender differences were found in the population we selected, which is in agreement with previous studies.

In our study, average position of mental foramen relative to the apex of second premolar was found to be 47% on the mesial side which is analogous to the findings by Phillips *et al.* (3,4) however Moiseiwitsch *et al.* (2) reported that 90% of foramina lied either at the second premolar or immediately mesial or distal to it. In our study, the vertical location of the foramen however varied drastically in the vertical plane (x/z standard deviation – 0.99 on left side and 1.12 on right side).

Results obtained from our study showed that size of mental foramen on left side was slightly larger than right side which is analogous to the study done by Phillips *et al.* (1). On the other hand, Yosue *et al.* (11,12) reported that there is no significant difference in the diameter of mental foramen.

Determining the morphological appearance and positional variation of MF is important for isolation of mental nerves and vessels when administering local anesthesia and performing surgeries. Because of its considerable clinical application, it is very important to know the normal range of possible locations of MF. The most frequent appearance of MF in our study was continuous type which showed variation from previous reports. The results of our study supports only few previous reported studies concerning the most frequent horizontal and vertical locations of MF, which clearly indicates that it has positional variations in different population groups. We therefore stress the importance of accurate radiographic identification of MF and interpretation before administration of local anesthesia or conducting any surgery of mandible in the vicinity of MF. These findings can be used as reference material by the dental practitioners of South India while performing clinical procedures that involve MF.
